# The Bacterial Diversity of Spontaneously Fermented Dairy Products Collected in Northeast Asia

**DOI:** 10.3390/foods10102321

**Published:** 2021-09-29

**Authors:** Zhongjie Yu, Chuantao Peng, Lai-yu Kwok, Heping Zhang

**Affiliations:** 1 Key Laboratory of Dairy Biotechnology and Engineering (Inner Mongolia Agricultural University), Ministry of Education, Inner Mongolia Agricultural University, Hohhot 010018, China; wayuzhongjie@163.com (Z.Y.); chuantaopeng@163.com (C.P.); Kwok_ly@yahoo.com (L.-y.K.); 2 Key Laboratory of Dairy Products Processing, Ministry of Agriculture and Rural Affairs, Inner Mongolia Agricultural University, Hohhot 010018, China; 3 Inner Mongolia Key Laboratory of Dairy Biotechnology and Engineering, Inner Mongolia Agricultural University, Hohhot 010018, China

**Keywords:** spontaneously fermented dairy product, microbial diversity, PacBio SMRT sequencing, geographical origin

## Abstract

Spontaneously fermented dairy products have a long history, and present diverse microorganisms and unique flavors. To provide insight into the bacterial diversity, 80 different types of spontaneously fermented dairy product samples’ sequence data that were downloaded from MG-RAST and NCBI and 8 koumiss and 4 shubat were sequenced by the PacBio SMRT sequencing platform. All samples including butter, sour cream, cottage cheese, yogurt, koumiss, shubat, and cheese, were collected from various regions in Russia, Kazakhstan, Mongolia and Inner Mongolia (China). The results revealed that Firmicutes and Proteobacteria were the most dominant phyla (>99%), and 11 species were identified with a relative abundance exceeding 1%. Furthermore, *Streptococcus salivarius*, *Lactobacillus helveticus*, *Lactobacillus delbrueckii*, *Enterobacter xiangfangensis*, and *Acinetobacter baumannii* were the primary bacterial species in the fermented dairy product samples. Principal coordinates analysis showed that koumiss and shubat stood out from the other samples. Moreover, permutational ANOVA tests revealed that the types of fermented dairy products and geographical origin significantly affected microbial diversity. However, different processing techniques did not affect microbial diversity. In addition, results of hierarchical clustering and canonical analysis of the principal coordinates were consistent. In conclusion, geographical origin and types of fermented dairy products determined the bacterial diversity in spontaneously fermented dairy product samples.

## 1. Introduction

Spontaneously fermented milk products have been produced and consumed for thousands of years. About 5000 BC, in the Mesopotamian plains, nomadic communities stored milk in stone pots or bags made from animal skins, leading to the production of the first spontaneously fermented milk. Subsequently, fermented milk was introduced into East Asia, including Russia, China, and Mongolia, by Tartars and Huns via the “Silk Road” [1]. Fermented milk is considered an essential part of the global diet. According to Euromonitor statistics, the fermented milk market exceeded 119 billion yuan in China in 2017, and it is expected to increase continuously over the coming years [2]. One reason for the substantial popularity of fermented milk products is their nutritional and functional benefits. Elie Metchnikoff consumed fermented milk as part of his diet, which was likely to be important for longevity [3]. Laboratory tests recently revealed that koumiss could modulate gut microbiota and increase plasma high-density cholesterol, which can be used to treat patients suffering from hyperlipidemia [4].

Traditionally, the production of spontaneously fermented dairy products relies on co-fermentation with various lactic acid bacteria (LAB) and yeast, resulting in various unique flavors and nutrients [5,6]. It is essential to understand how the microbial communities in fermented dairy products influence their unique physio-chemical and sensory properties. Therefore, it is crucial to characterize their microbial structure and the factors that shape the microbial communities. Conventional culture-dependent methods have been used to identify and describe the microbial composition in fermented milk products. However, the identified microbial diversity has not been comprehensively described due to the difficulty in simulating natural growth conditions. With the developments of molecular biology, scientists are learning more about the composition of microorganisms in spontaneously fermented dairy products. Motato et al. and Lappa et al. used Denatured Gradient Gel Electrophoresis (DGGE) and matrix-assisted laser desorption/ionization time-of-flight mass spectrometry (MALDI-TOF MS) technology respectively, and characterized the composition of microorganisms of Suero Costeño, a kind of spontaneously fermented dairy product from Kolumbia, and Staka, a naturally fermented cream produced from sheep or mixtures of sheep and goat milk in Greece [7,8].

With the continuous development of bioinformatics and biotechnology, the utilization of high-throughput DNA sequencing has become more prevalent due to its cost-efficiency. Several studies have analyzed the microbiota of fermented dairy products using second-generation sequencing techniques. Zhong et al. (2016) examined the bacterial compositions of 85 spontaneously fermented milk samples from China, Mongolia, and Russia using a second-generation sequencing technique. The results indicated that the geographic origin and sample type influence the microbial diversity of these products [9]. However, the second-generation DNA sequencing technology exhibits a significant drawback because of short read length and low taxonomic resolution. Recently, Pacific Biosciences (PacBio, Menlo Park, CA, USA) developed single-molecule real-time sequencing technology (SMRT) coupled with full-length 16S rRNA gene sequencing, which offers a considerable advantage in generating long reads, allowing a high taxonomic resolution to the species level. This technique has been widely applied in evaluating microbial communities in spontaneously fermented foods, such as tibicos [10] and paocai [11].

**Figure 1:**
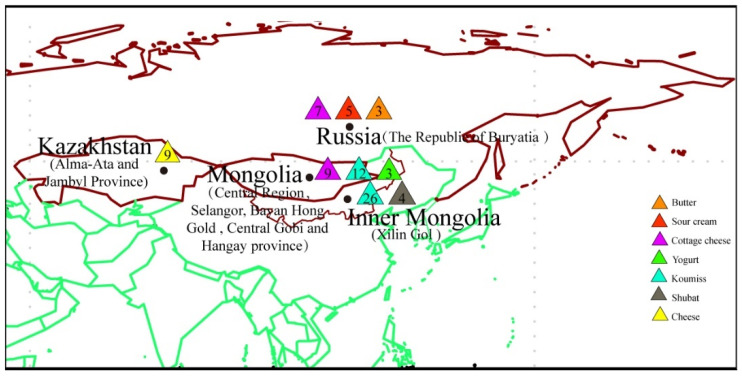
Geographic locations and sample types. Eight different spontaneously fermented dairy products were collected from Mongolia, Russia, Kazakhstan, and Chinese Inner Mongolia.

In summary, the spontaneously fermented dairy products exhibit an abundance of microbial resources, and may contain a substantial amount of lactic acid bacteria and probiotics. However, previous studies were either limited to a certain type or region of spontaneously fermented dairy products, or could not fully clarify the microbial diversity. In this study, eight types of koumiss and four types of shubat were collected from Mongolia and Inner Mongolia in China. The full-length 16S rRNA gene data of the flora of 80 spontaneously fermented dairy products sequenced via the PacBio SMRT sequencing platform, and collected from Mongolia, Russia, Kazakhstan, and Chinese Inner Mongolia, were downloaded. In total, 92 spontaneously fermented dairy products collected from 4 different countries (Figure 1, Table 1) accounted for almost all such products commonly consumed by the nomadic people living in Northeast Asia. All sampling positions were located in the Mongolian Plateau, and had similar climatic conditions—temperate continental climate. The third-generation DNA sequencing technology represented by the PacBio SMRT sequencing platform could completely and unbiasedly reveal the microbial diversity of the samples.

## 2. Materials and Methods

### 2.1. Data and Sample Collection

This study reanalyzed data from 80 previously collected spontaneously fermented dairy product samples using the PacBio SMRT sequencing platform. Here, 14 jiaoke (a kind of fermented butter produced in Inner Mongolia Xilin Gol) (sequence data downloaded from MG-RAST, Project ID: mgp92388), 9 cheese (sequence data downloaded from NCBI, Project ID: PRJNA347428, collected from Alma-Ata and Jambyl provinces of Kazakhstan), 7 cottage cheese, 3 butter, and 5 sour cream (sequence data downloaded from MG-RAST, Project ID: mgp83644, collected from The Republic of Buryatia), 14 koumiss (sequence data downloaded from MG-RAST, Project ID: 91551, collected from Inner Mongolia Xilin Gol), and 16 koumiss, 9 cottage cheese, and 3 yogurt (sequence data downloaded from MG-RAST, Project ID: mgp87678: 4 koumiss and 3 yogurt collected from Mongolian Bayan Hong Gold province, 9 cottage cheese collected from Mongolian Hangay and Selangor province, 12 koumiss collected from Inner Mongolia Xilin Gol). We collected 8 koumiss and 4 shubat (a kind of fermented camel milk) from Mongolian Hangay and Central Gobi province, and Inner Mongolia Xilin Gol sequenced by the same sequencing platform (sequence data uploaded to MG-RAST, Project ID: mgp96817).

### 2.2. DNA Extraction, PCR Amplification, and PacBio SMRT Sequencing

Koumiss and shubat were obtained from Mongolia and Inner Mongolia. The samples were collected using a sterile spoon, stored in 50 mL sterile centrifuge tubes, and sent to our laboratory packed on ice. Total DNA was extracted by the Omega DNA isolation kit (R6731-01, E.Z.N.A^TM^, Omega Bio-Tek, Norcross, GA, USA). The quality of DNA was checked by agarose gel electrophoresis on a 0.8% gel and spectrophotometry (Thermo Fisher Scientific, Waltham, MA, USA). The full-length region of 16S rRNA genes was amplified by the forward primers 27F (5′-GAGAGTTTGATCCTGGCTCAG-3′) and the reverse primers 1541R (5′-AAGGAGGTGATCCAGCCGCA-3′) with the PCRBIO Taq DNA polymerase (PCR Biosystems Ltd., London, UK). The primers contained 16-base barcodes to distinguish between samples. The PCR programs were as follows: 95 °C for 5 min, then 30 cycles at 95 °C for 30 s, 58 °C for 45 s, and 72 °C for 1 min, with a final extension of 72 °C for 7 min (Hou et al., 2019). The quality of the PCR products were checked by the Agilent DNA 1000 Kit and an Agilent 2100 Bioanalyzer (Agilent Technologies, Santa Clara, CA, USA). PCR products were electrophoresed in a 1.2% agarose gel and were used for constructing DNA libraries with the Pacific Biosciences Template Prep Kit 2.0 (Pacific Biosciences). Sequencing was performed using P6-C4 chemistry on a PacBio RS II platform (Pacific Biosciences, Menlo Park, CA, USA) in accordance with the manufacturer’s instructions. Quality control for PCR and sequence preprocessing was performed as described previously [12].

### 2.3. Bioinformatics Analysis

The amplified full-length 16S rRNA genes were processed according to the following criteria: (i) A minimum of up to five full passes (ii) a minimum predicted accuracy of 90 (iii) a minimum of a 1400 read length of inserts and (iv) a maximum of a 1800 read length of inserts in the SMRT Portal version 2.3 (Pacific Biosciences, Menlo Park, CA, USA) of the protocol RS_ReadsOfinsert.1.

The QIIME [13] (version 1.7) was applied to perform bioinformatics analyses. In summary, PyNAST [14] and UCLUST [15] were used to align the sequences under 100% clustering of sequence identity. The unique sequence set was classified into operational taxonomic units (OTUs) under a 97% identity threshold using UCLUST after selecting the representative sequences. The potential chimeric sequences in the representative set of OTUs were removed using ChimeraSlayer [16]. Three databases, namely Ribosomal Database Project [17] (RDP, Release 11.5), Greengenes [18] (version 13.8), and Silva [19], were applied to assign the phylogeny of the OTU representative sequence at a minimum bootstrap threshold of 80% [20]. The OTU table was subsampled according to an adjusted sampling depth of all samples using the multiple_rarefactions.py program in the QIIME pipeline. A de novo taxonomic tree was constructed, employing a representative chimera-checked OTU set in FastTree [21] for calculating alpha and beta diversity. Shannon–Wiener, rarefaction estimators, Simpson, and Chao1, were calculated to evaluate the α-diversity. The UniFrac distance was based on the phylogenetic tree [22] to evaluate the β-diversity. Both weighted and unweighted calculations were performed for principal coordinates analysis (PCoA).

### 2.4. Statistical Analyses

Statistical analyses were performed by using R packages (http://www.r-project.org/ accessed on 10 August 2021). Differences between the microbial population groups were evaluated using the Kruskal–Wallis test. The permutational ANOVA (PERMANOVA) test was performed to detect the effect of geographical origin, types of fermented dairy products, and production methods on the bacterial diversity in the spontaneously fermented dairy products.

## 3. Results

### 3.1. Bacterial Alpha Diversity Analysis

**Figure 2:**
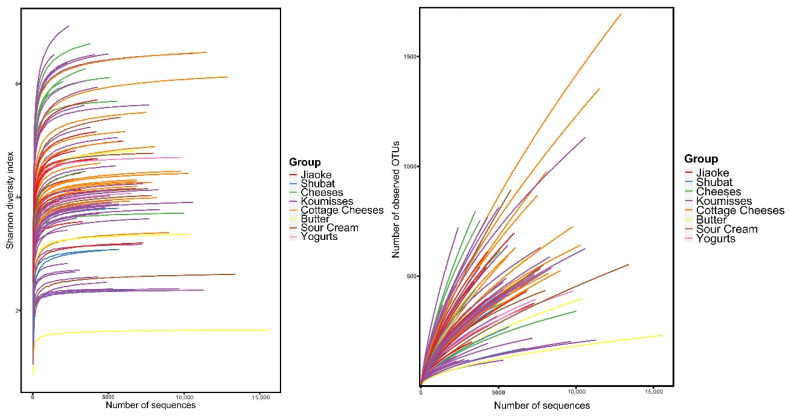
Rarefaction and Shannon diversity index curves for the spontaneously fermented dairy product samples.

The raw data contained a total of 573,992 reads from the 92 naturally fermented dairy product samples, ranging from 1804 to 157,883, with an average of 6239 ± 2864 reads for each sample. A total of 17,558 OTUs were obtained at a 97% similarity level, ranging from 114 to 1696, with an average of 486 ± 253OTUs for each sample. Each OTU was assigned to the lowest taxonomic level based on information extracted from the RDP, Greengene, and Silva databases, after which the relative abundance of each taxon was determined. On average, 2.15% and 8.30% of the sequences were not classified into genus and species levels, respectively. The Shannon diversity curves of all the samples reached the saturation phase, while the rarefaction curves did not reach this stage (Figure 2). These results suggested that new phylotypes could be discovered if the sequencing depths were increased. The current sequence depth allowed for the capturing of most phylotypes.

In the present study, the Shannon and Simpson diversity indices were applied to calculate bacterial diversity, and the Chao1 index and the number of observed species were utilized to measure bacterial richness (Table 2). In order to facilitate comparison, each sample sequence was normalized randomly to 1410 sequences/sample. The differences in bacterial diversity were significant in all types of spontaneously fermented dairy products. Cheese had the highest microbial diversity indexes, followed by yogurt and cottage cheese, and the lowest diversity was observed in the butter samples. Moreover, cheese exhibited the highest bacterial richness index among all the samples, and shubat exhibited the lowest richness. The results showed a substantial difference between the diversity and richness of different types of spontaneously fermented dairy product samples.

### 3.2. Composition of the Bacterial Structure in Spontaneously Fermented Dairy Products

**Figure 3:**
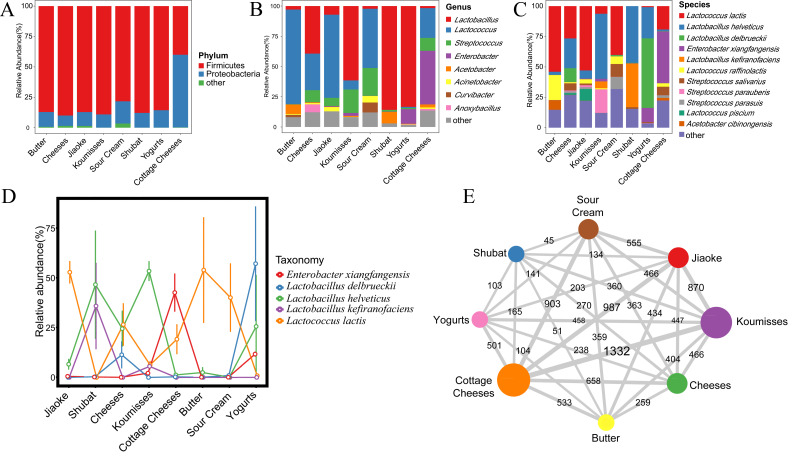
The average compositions of spontaneously fermented dairy product samples at the phylum (**A**), genus (**B**), and species (**C**) levels. Differential abundant species between different spontaneously fermented dairy product samples (**D**). The OTU network map between the pairs of spontaneously fermented dairy products (**E**). The size of the circle represents the number of OTUs, and the thickness of the line between the circles and the number on the line represent the number of OTUs shared between each pair of samples. ‘Others’ refer to taxa that had <1% overall relative abundance.

To analyze the bacterial composition of each sample, the sequences of 92 spontaneously fermented dairy product samples were analyzed using the PacBio SMRT sequencing platform and were classified to the phylum, genus, and species levels. At the phylum level, 22 phyla were identified from the samples. The relative abundance of Firmicutes and Proteobacteria exceeded 99% (Figure 3A). The content of Proteobacteria exceeded that of Firmicutes only in the cottage cheese samples. Samples from Mongolia contained significantly higher Proteobacteria (*p* = 0.001) levels and lower Firmicutes (*p* = 0.001) levels.

Furthermore, 342 bacterial genera were identified. Seven genera displayed an average relative abundance exceeding 1%, namely *Lactobacillus*, *Lactococcus*, *Streptococcus*, *Enterobacter*, *Acetobacter*, *Pseudomonas,* and *Acinetobacter*, accounting for 90.18% (Figure 3B). 

Since the PacBio SMRT sequencing platform can cover the full read length of the 16S rRNA gene (Mosher et al., 2014), the relative abundance at the species level was resolved. The relative abundances of 11 species exceeded 1%, accounting for 81.37% of the total microbiota population, and included *Lactobacillus helveticus*, *Lactococcus lactis*, *Enterobacter xiangfangensis*, *Streptococcus parauberis*, *Lactobacillus kefiranofaciens*, *Lactobacillus delbrueckii*, *Streptococcus salivarius*, *Lactococcus raffinolactis*, *Lactococcus piscium*, *Streptococcus parasuis,* and *Acetobacter cibinongensis* (Figure 3C). It displayed distinct bacterial structures among the different types of naturally fermented dairy products (Figure 3D). Compared with other types of spontaneously fermented milk, *L. delbrueckii* (*p* < 0.001), *E. xiangfangensis* (*p* < 0.001), and *L. kefiranofaciens* (*p* < 0.001) were predominant in yogurt, cottage cheese, and shubat, respectively. The abundance of *L. lactis* exceeded 40% in jiaoke, sour cream, and butter. The content of *L. helveticus* in koumiss surpassed the levels of other bacterial species, while *Lac. lactis, L. helveticus,* and *L. delbrueckii* dominated in cheese.

Finally, the data were analyzed on an OTU level to examine the configuration of spontaneously fermented dairy product samples. As mentioned above, 17,558 OTUs were identified in the samples, revealing 10 OTUs in all 8 types of spontaneously fermented dairy products, namely *S. salivarius*, *L. helveticus*, *L. delbrueckii*, *E. xiangfangensis,* and *Acinetobacter baumannii.* Therefore, it was inferred that these five species represented the primary bacteria in the spontaneously fermented dairy products. A network map was constructed based on the OTUs shared between the pairs of spontaneously fermented dairy products (Figure 3E). Cottage cheese and koumiss shared the maximum number of OTUs, followed by cottage cheese and jiaoke, while shubat and sour cream shared the least number of OTUs.

### 3.3. Bacterial Structures of the Spontaneously Fermented Dairy Products

**Figure 4:**
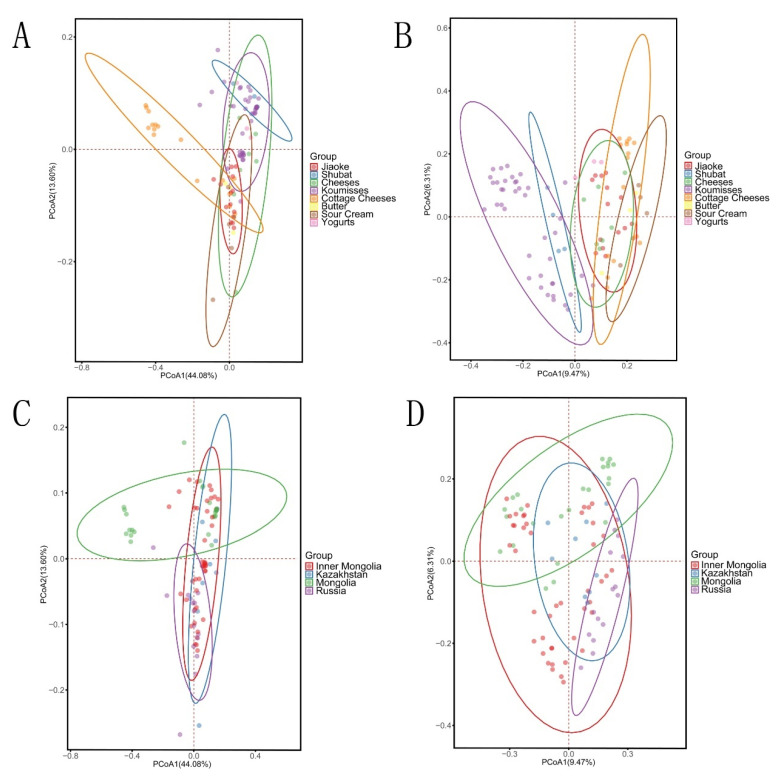
PCoA plots based on the weighted (**A**) and unweighted (**B**) UniFrac distances of the different types of dairy products and the weighted (**C**) and unweighted (**D**) UniFrac distances of the different geographical origins.

The bacterial structures of the spontaneously fermented dairy product products were analyzed regarding different types of fermented dairy product and different geographical origins using PCoA. First, PCoA was performed based on weighted and unweighted UniFrac distances (Figure 4A,B). Some clustering overlaps were found in eight different types of milk. Data points were separated into weighted (principal components 1 (PC1) and principal components 2 (PC2), accounting for 44.08% and 13.60% of the total variance, respectively) and unweighted (PC1 and PC2, accounting for 9.47% and 6.31% of the total variance, respectively) UniFrac during PCoA. The koumiss and shubat samples were clearly distinguished from other samples in unweighted UniFrac. PCoA indicated an abundance of the low-level *Serratia grimesii* and *Paenibacillus odorifer* species in koumiss and shubat only. The high-level species, *E. xiangfangensis* (43.67%, *p* < 0.001), was abundant in the cottage cheese sample, clearly distinguishing cottage cheese from other samples. These results indicated that bacterial composition was possibly associated with different types of spontaneously fermented dairy products.

Regarding geographical origin, no distinct clustering was found in the unweighted principal component scores plot (Figure 4C). However, analysis of the weighted principal components showed nine outliers due to the high abundance of *E. xiangfangensis* in the cottage cheese samples collected from Mongolia (Figure 4D).

**Figure 5:**
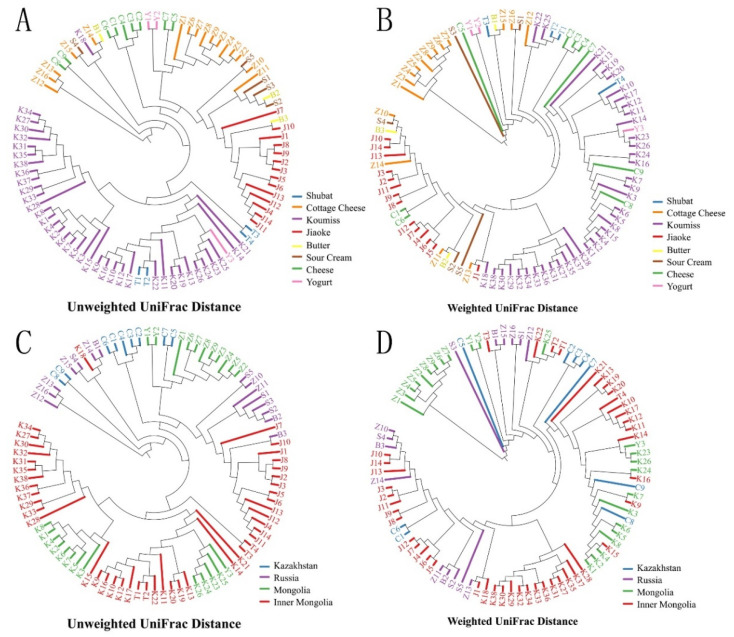
Hierarchical clustering analysis based on the unweighted and weighted UniFrac distances of different fermented dairy product types (**A**,**B**) and geographical origins (**C**,**D**) via the unweighted pair group method with arithmetic means (UPGMA).

Furthermore, PERMANOVA tests were performed using UniFrac distance to determine the factors influencing the bacterial diversity of spontaneously fermented dairy products. First, to evaluate the influence of various types of fermented dairy products on bacterial diversity, 12 koumisses, 9 kinds of cottage cheese, and 3 yogurts were collected from Mongolia, and 14 jiaoke and 25 koumisses were collected from Inner Mongolia. Significant differences were apparent between the unweighted (*p* < 0.001 in Mongolia and Inner Mongolia) and weighted UniFrac distances (*p* < 0.001 in Mongolia and Inner Mongolia) of these samples. Next, the cottage cheese and koumiss samples were used to assess the impact of different geographical origins on bacterial diversity. Significant differences in bacterial diversity were observed between the 16 types of cottage cheese (*p* < 0.001 in unweighted and weighted UniFrac distance) collected from Mongolia (9) and Russia (7). Similar results were found in 40 koumisses (*p* < 0.001 in unweighted and weighted UniFrac distance) collected from Mongolia (12) and Inner Mongolia (28). Moreover, this study also evaluated the impact of different processing methods on the bacterial diversity in three kinds of butter, five sour creams, and seven types of cottage cheese, which were processed using cow’s milk and collected in Russia. No significant differences were found among these samples (*p* = 0.0076 in unweighted UniFrac distance and *p* = 0.27 in weighted UniFrac distance). Additionally, the UniFrac distance was used to perform hierarchical clustering in the unweighted pair group method with arithmetic mean (UPGMA) to observe the evolutionary distance among samples according to the distance of the branches and clusters (Figure 5A–D). For the different fermented dairy product types, different clusters were formed in two types of UniFrac distance. Particularly, in unweighted UniFrac distance, clusters of koumisses and Jiaoke almost contained their own samples. Furthermore, for some types of fermented dairy products dispersed in the dendrogram, such as yogurt and butter, this could be attributed to the limited sample size. Although the clustering did not show differences between the fermented dairy products from different geographical regions, some subclusters of specific sample collection sites were still formed.

**Figure 6:**
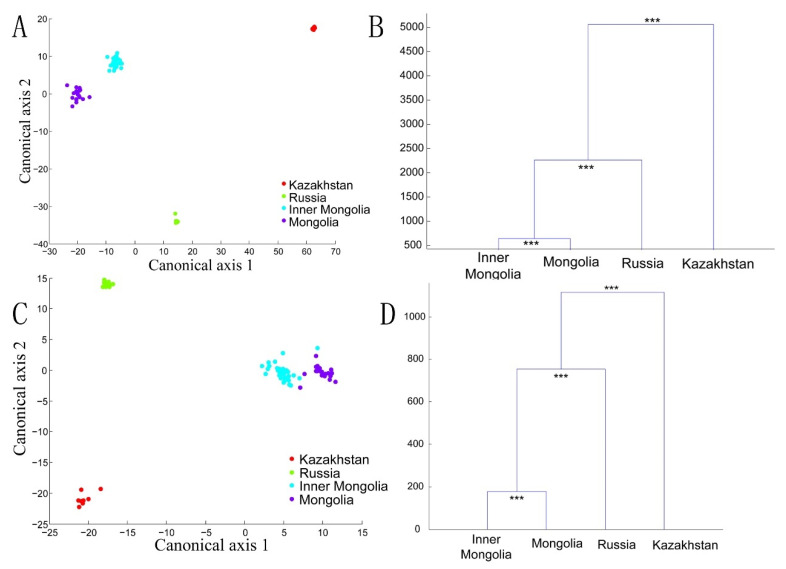
CAP and cluster analysis of the spontaneously fermented dairy products’ microbiota communities based on the unweighted (**A**,**B**) and weighted (**C**,**D**) UniFrac distances. The Mahalanobis distances of the fecal bacterial communities generated via CAP were used for the cluster analysis. Significant differences are illustrated as *** (*p* < 0.001) (**B,D**).

**Figure 7:**
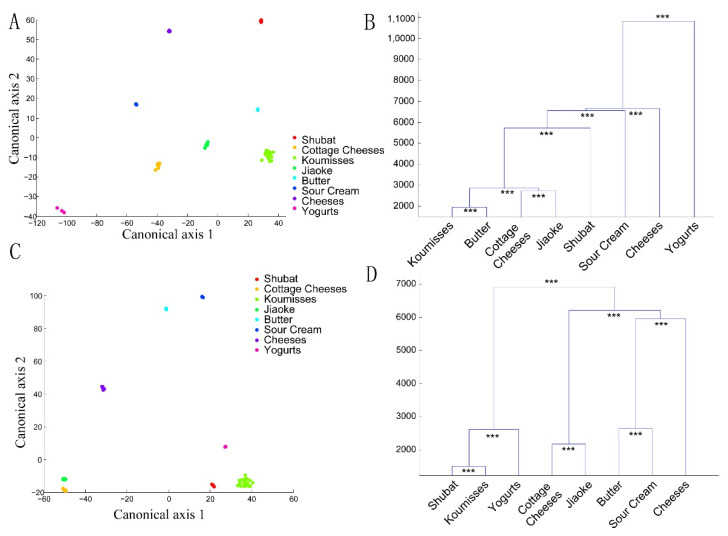
CAP and cluster analysis of the spontaneously fermented dairy products microbiota communities based on the unweighted (**A**,**C**) and weighted (**B**,**D**) UniFrac distances. The Mahalanobis distances of the fecal bacterial communities generated via CAP were used for the cluster analysis. Significant differences are illustrated as *** (*p* < 0.001) (**B,D**).

A canonical analysis of principal coordinates (CAP) based on 85% of the principal components calculated using PCoA analysis was performed to compare the microbiota of different geographical areas (Figure 6A–D) and types of milk (Figure 7A–D). The dendrograms showed significant differences between the samples in two types of UniFrac distances. These results revealed that the type of fermented dairy product and geographical origin both shaped the bacterial diversity of spontaneously fermented dairy products.

## 4. Discussion

Spontaneously fermented dairy products have a long history and contain valuable microbial resources. Using the PacBio SMRT sequencing platform, this study analyzed the full length of the 16S rRNA gene data of 92 spontaneously fermented dairy product samples collected from various regions in Russia, Kazakhstan, Mongolia, and Inner Mongolia from 2015 to 2019. The reanalysis aimed to provide more precise insights into the bacterial diversity at the species level in spontaneously fermented milk in Northeast Asia. After the analysis, the complete dataset contained 573,992 reads and 17,558 OTUs derived from the 92 samples. Cheese displayed the highest Chao1, Shannon, and Simpson indices among all the samples, implying that it had the most diverse and richest bacterial compositions. The processing procedures of cheese from Kazakhstan contain milk collection, pasteurization, the addition of the starter (natural whey), squeezing, splitting, and seasoning. During complex manufacturing processes, thousands of bacteria and fungi are inoculated into cheese and cottage cheese and they are especially prevalent during the final seasoning procedure. As shown in Table 2, the bacterial diversity and richness in cottage cheese ranked second. Several researchers revealed that fungi in fermented products, such as cheese, may promote the growth of certain bacteria. Several bacterial species, such as *Corynebacterium*, *Halomonas*, *Pseudomonas*, *Pseudoalteromonas*, and *Vibrio,* have exhibited poor growth in the absence of a fungal partner [23]. Moreover, jiaoke and sour cream had a similar production process. Jiaoke, similar to sour cream, has the same white color, fragrant flavor, and delicious taste. Therefore, jiaoke and sour cream displayed a similar number of OTUs, 555 (Figure 3E), and in terms of bacterial composition, they predominantly contained *Lac. lactis* and relatively low levels of *L. helveticus*, *L. delbrueckii*, *E. xiangfangensis,* and *L. kefiranofaciens* (Figure 3C).

Regarding bacterial composition, 698 bacterial species were identified in the samples. Although these species were unevenly distributed in various fermented dairy products samples, *S. salivarius*, *L. helveticus*, *L. delbrueckii*, *E. xiangfangensis,* and *A. baumannii* were identified in all the specimens, accounting for 39.72%. Of these five species, *A. baumannii* is a potential pathogen that causes ventilator-associated pneumonia (VAP), urinary tract infections, and bacteremia in intensive care units (ICUs) [24]. However, it only accounted for 0.22% of the average relative abundance, which is unlikely to pose health problems. Furthermore, *Acinetobacter* was also detected in fermented dairy products. Gao et al. (2013) and Parker et al. (2018) detected *Acinetobacter* in Tibetan kefir grains and *lait caillé* [25,26]. Moreover, Hou et al. (2015) and Araújo et al. (2015) found *Acinetobacter* in infant formula using culture-dependent and culture-independent methods [20,27]. *S. salivarius* and *E. xiangfangensis* are Gram-positive LAB [28]. In this study, *S. salivarius* was detected in cheese, jiaoke, sour cream, shubat, and cottage cheese samples, with a relative abundance exceeding 1%. Besides being typical commensal bacteria in the mouths of infants and adults, *S. salivarius*, *S. vestibularis,* and *S. thermophiles* are genetically similar [29,30,31], and the high level of 16S rRNA sequences can lead to misclassification [32]. *S. salivarius* and *E. xiangfangensis* were predominant in the cottage cheese samples collected from Mongolia. However, the same type of fermented dairy product collected from Russia displayed a significantly low percentage (74.10% vs. 2.25%) [26,33]. On an industrial level, fermented dairy products are typically produced by starter culture, which is inoculated into sterilized raw milk. However, the sources for spontaneously fermented milk starter cultures vary extensively. For example, bacteria can propagate from vessels or raw milk [1], and some complex microbial communities are also used in back-slopping [26], such as during the production of kefir [33]. Additionally, the starter culture may be exchanged between friends and relatives, causing the naturally fermented dairy product in certain regions to display similar microorganism characteristics.

*Lactobacillus* is the predominant genus in naturally fermented dairy products [34,35]. Using pyrosequencing technology, Zhong et al. (2016) found that *Lactobacillus*, *Streptococcus*, and *Lactococcus* were the predominant genera in 85 naturally fermented milk products [9]. In this study, *Lactobacillus* was the highest relatively abundant genus in all samples. *L. helveticus,* *L. kefiranofaciens,* and *L. delbrueckii* were widely present as the primary bacterial species in the fermented dairy product samples. Besides *Lactobacillus*, *Lactococcus,* and *Streptococcus*, species such as *Lac. lactis*, *Lac. raffinolactis*, *S. salivarius,* and *S. parauberis* were also highly abundant in fermented dairy products. Previous studies have shown that different types of fermented dairy products from different locations exhibited substantial bacterial diversity. Gsudu et al. (2016) and Yahya et al. (2017) observed that *L. helveticus*, *L. kefiranofaciens,* and *S. parauberis* were the dominant species in koumiss and shubat [36,37]. *L. delbrueckii* is typically used to produce yogurt [38] and accounted for more than half of the average relative abundance in this product [39]. *Lac. lactis* represented more than 40% in the above two sample types, and it was speculated that its lipid metabolism capacity makes it adaptable to high-fat environments [40]. Compared with commercially fermented milk, there is no heat-treatment step during the processing of fermented milk products. The presence of LAB is crucial since it can convert lactose to lactic acid during the fermentation process, enhancing the acidity in milk while producing peroxide and bacteriocins, which can suppress the growth of pathogens [39,41]. In addition, LAB can also produce other metabolites, such as exopolysaccharides, B vitamins, and bioactive peptides that can enhance the rheological properties and sensory perception of fermented milk, preventing several disorders, such as hypertension [42,43].

This study focused on the factors that determined the bacterial diversity of naturally fermented dairy products. PCoA, PERMANOVA, hierarchical clustering, and CAP analyses revealed significant variations in the naturally fermented dairy products’ microbiota structures across sampling sites and fermented dairy products. These results were consistent with previous studies, which found that geographical origin could influence the bacterial structure of naturally fermented dairy products [9,36,37]. However, no substantial differences were found between fermented milk types [9], which could be ascribed to the number of sample types. Other than sampling sites and fermented milk types, this study also evaluated the impact of manufacturing processes using samples from the same collection locations and the same types of milk, but no significant differences were observed. Although not the focus of this study, hygiene conditions also played an important role. Walsh et al. found that a hygienic environment during production significantly influenced the traditional Ghanaian fermented milk product Nunu’s bacterial structure [44].

In conclusion, this work provides insight into the bacterial structure of 92 spontaneously fermented dairy product samples, including jiaoke, cheese, koumiss, cottage cheese, yogurt, butter, sour cream, and shubat, collected from Russia, Kazakhstan, Mongolia, and Inner Mongolia. These samples were sequenced using the PacBio SMRT sequencing technique. Furthermore, this study characterizes the bacterial configuration, indicating that geographical origins and type of fermented dairy product are both essential in shaping the bacterial diversity of naturally fermented dairy products.

## Figures and Tables

**Table 1:** Sample information.

Types of Sample	Sample ID	Number of Samples	Sampling Location
Jiaoke	J1-J14	14	Inner Mongolia
Cheese	C1-C9	9	Kazakhstan
Cottage cheese	Z10-Z16	7	Russia
Butter	B1-B3	3	Russia
Sour cream	S1-S5	5	Russia
Koumiss	K9-K22	14	Inner Mongolia
Koumiss	K27-K38	12	Inner Mongolia
Koumiss	K23-K26	4	Mongolia
Koumiss	K1-K8	8	Mongolia
Shubat	T1-T4	4	Inner Mongolia
Yogurt	Y1-Y3	3	Mongolia
Cottage cheese	Z1-Z9	9	Mongolia

**Table 2:** The alpha diversity in the spontaneously fermented dairy product samples.

	Item	Jiaoke	Cheeses	Koumisses	Cottage Cheeses	Yogurts	Butter	Sour Cream	Shubat	*p*-Value ^1^
Index										
Chao1		507.55 ± 200.59	758.50 ± 389.64	542.44 ± 318.33	661.16 ± 274.07	392.04 ± 99.49	351.97 ± 244.08	608.39 ± 375.97	333.53 ± 138.22	0.18
Observed species		188.91 ± 52.51	276.13 ± 104.95	204.98 ± 97.15	217.75 ± 55.18	160.26 ± 12.01	124.96 ± 69.84	189.86 ± 68.03	127.83 ± 45.33	0.03
Shannon		4.33 ± 0.61	5.38 ± 1.03	4.11 ± 1.30	4.43 ± 0.73	4.35 ± 0.39	3.13 ± 1.50	3.99 ± 0.89	2.96 ± 0.55	0.01
Simpson		0.80 ± 0.07	0.90 ± 0.07	0.77 ± 0.12	0.82 ± 0.07	0.87 ± 0.05	0.64 ± 0.26	0.75 ± 0.16	0.67 ± 0.09	0.01

Note: Results are expressed as mean ± SD. ^1^ Kruskal–Wallis test was used.

## Data Availability

The sequence data of koumiss and shubat is uploaded to MG-RAST, Project ID: mgp96817.

## The list of Abbreviations

Abbreviation	Full name
PCR	Polymerase Chain Reaction
OTU	Operational Taxonomic Unit
RDP	Remote Desktop Protocol
*L. delbrueckii*	*Lactobacillus delbrueckii*
*E. xiangfangensis*	*Enterobacter xiangfangensis*
*L. kefiranofaciens*	*Lactobacillus kefiranofaciens*
*L. lactis*	*Lactobacillus lactis*
*L. helveticus*	*Lactobacillus helveticus*
*Lac. raffinolactis*	*Lactococcus raffinolactis*
*Lac. lactis*	*Lactococcus lactis*
*S. salivarius*	*Streptococcus salivarius*
*S. parauberis*	*Streptococcus parauberis*
*S. vestibularis*	*Streptococcus vestibularis*
*A. baumannii*	*Acinetobacter baumannii*
